# Xanthoma Diabeticorum

**Published:** 1909-07-10

**Authors:** 


### Dermatology.

### XANTHOMA DIABETICORUM.

There is a rather uncommon, but when seen a very striking, skin eruption that occurs in glycosuric subjects and is known as xanthoma diabeticorum. It seems to have been first particularly described by Malcolm Morris; and although it has relations with xanthoma or xanthelasma due to other causes such as chronic jaundice, it has usually been regarded as sufficiently different to be distinctive. The case we are about to describe suggests that this is not really the case, and that although the eruption in diabetic subjects sometimes presents an appearance that will lead at once to suspicion of glycosuria, there are a certain number of other cases in which, though coinciding with glycosuria, the xanthoma has precisely the same appearance as has ordinary xanthelasma planum.

### Characteristics of the Eruption.

The variety which is regarded as typical of diabetic subjects is much rarer than are the forms termed xanthelasma palpebrarum, xanthelasma tuberosum, and xanthelasma planum. Besides the condition of the urine its special features are as follows. The yellow spots are raised in a conical or domed shape and they are surrounded by a pale, dull red zone. They may be mistaken for acne pustules, but on puncture they are found to be solid. They come out first upon the extensor surfaces of the limbs, particularly on the elbows, then on the lower part of the back and abdomen, and on the buttocks. They are also to be found on the genital organs, on the palms of the hands, the face, chin, neck, scalp, and exceptionally on the eyelids.

The only subjective symptom generally observed is itching, which is more marked when the eruption is on the wane than when it is either developing or in full swing. The mucous membrane of the mouth is occasionally affected. The lesions appear rapidly and take weeks to disappear. Fresh crops are produced after some time. The patients are mostly men, apparently in good health, but inclined to stoutness. As regards the constitutional state it may be stated as a general law that there is, has been, or will be sugar in the urine.

### An Illustrative Case.

In the following case, however, the xanthoma was far less of the acne-resembling form, and more like xanthelasma due to chronic jaundice. The patient was a well-to-do man aged 65. Four or five years previously he had experienced a troublesome balanitis and posthitis for which he had consulted doctors, with the result that glycosuria to the extent of 4.5 per cent. was detected. There were none of the other usual symptoms of diabetes mellitus—no thirst, no polyuria, no wasting. Under observation it was found that the glycosuria was not constant. In the course of time he noticed a skin lesion upon his right leg. He attributed its coming to the fact that he had had obscure pains in the limb, to cure which he had resorted to rubbings and thumpings. When it had been present a year it had only slowly enlarged. Situated upon the external aspect of the leg some four inches above the malleolus, it was made up of two plaques, an upper and a lower, united to one another by a narrow junction. The general tint of the patches was that of yellow ochre. At the edges the lesion terminated in a series of small flat papules, close to but separated from the main mass. The plaques were very slightly raised, firm, and resistant; the surrounding skin was quite soft and supple. There was no obvious vascular dilatation, but a little local hyperæsthesia and pruritus.

In the upper part of the same leg there was another patch of xanthelasma, less distinct than those below, composed of several scattered smaller patches of typical yellow-ochre colour. Nowhere did the patient exhibit xanthelasma tuberosum. The only other parts of the body upon which the lesion could be found were the palms of the hands and the soles of the feet; here one could see definite lines in the skin of a diffuse sulphur yellow colour, nearly but not quite corresponding with the natural lines or sulci in these parts. The eyelids were unaffected.

It should be noted that, except for the occasional glycosuria and for the xanthoma the patient was, and always had been, perfectly sound. He had never been jaundiced, nor had he suffered from any symptom of gall-stones or any other hepatic disease. The urine contained neither bile pigments nor urobilin. There seems no doubt that he is an instance of xanthoma diabeticorum conforming rather to the xanthoma planum type than to that which has sometimes been regarded as distinctive of diabetes.

### Pathology and Prognosis.

The skin eruption, whether of the eyelids or of the multiplex or "diabetic" variety, is generally looked upon as an inflammatory neoplastic process. There is an aggregation of cells resembling those met with in atheroma of arteries, which are formed partly from leucocytes, partly from connective tissue corpuscles. Instead of softening and breaking down into pus, these cells undergo a peculiar fatty change, which results in the production of a firm yellow lipochrome or fat-pigment, such as is always present in muscles undergoing fatty degeneration, and which is the cause of the distinctive yellow colour of xanthoma.

Prognosis is not materially affected by the occurrence of the eruption. Indeed, the cases of diabetes in which it occurs are rather those of the mild than of the severe type. Xanthoma may be the first evidence of glycosuria. Sometimes the glycosuria and the xanthomatous eruption alternate with one another, so that when the skin lesion comes out glycosuria disappears and *vice versa*.

It is important, therefore, to examine the urine in all cases of xanthoma or xanthelasma; and if sugar is not present in it at one examination, fresh specimens should be tested from time to time.

